# Supplementary material for: Measuring Comprehensive, General Health Literacy in the General Adult Population: The Development and Validation of the HLS19-Q12 Instrument in Seventeen Countries
Source: Int J Environ Res Public Health. 2022 Oct 29;19(21):14129. doi: 10.3390/ijerph192114129 (PMC9659295; doi:10.3390/ijerph192114129)
Supplement: Supplementary file 1 [file ijerph-19-14129-s001.zip › Supplementary_TableS2.pdf]

Table S2. Table Standardized Coefficients Single-Factor CFA of the polytomous items.

Items	AT	BE	BG	CH	CZ	DE	DK	FR	HU	IE	IL	IT	NO	PT	RU	SI	SK	Mean
1	0.61	0.67	0.63	0.53	0.70	0.54	0.65	0.59	0.49	0.58	0.63	0.67	0.60	0.78	0.71	0.72	0.71	0.64
2	0.62	0.62	0.68	0.67	0.68	0.61	0.62	0.68	0.62	0.56	0.68	0.77	0.60	0.75	0.77	0.79	0.72	0.67
3	0.59	0.68	0.73	0.63	0.64	0.62	0.69	0.71	0.66	0.54	0.70	0.72	0.54	0.70	0.77	0.65	0.70	0.66
4	0.60	0.72	0.67	0.60	0.58	0.52	0.70	0.62	0.67	0.65	0.67	0.66	0.70	0.71	0.73	0.77	0.65	0.66
5	0.65	0.62	0.67	0.69	0.63	0.61	0.66	0.74	0.54	0.59	0.70	0.68	0.59	0.70	0.74	0.76	0.73	0.66
6	0.71	0.75	0.22	0.65	0.64	0.64	0.67	0.75	0.73	0.71	0.74	0.75	0.71	0.79	0.77	0.75	0.73	0.69
7	0.64	0.70	0.70	0.60	0.58	0.60	0.67	0.77	0.70	0.64	0.68	0.74	0.68	0.84	0.77	0.82	0.69	0.70
8	0.59	0.56	0.58	0.51	0.66	0.55	0.62	0.70	0.53	0.46	0.73	0.69	0.50	0.73	0.75	0.53	0.64	0.61
9	0.69	0.73	0.69	0.64	0.55	0.60	0.72	0.71	0.74	0.64	0.66	0.67	0.67	0.89	0.75	0.78	0.76	0.70
10	0.59	0.63	0.66	0.52	0.54	0.38	0.59	0.63	0.69	0.64	0.65	0.66	0.63	0.82	0.68	0.70	0.62	0.63
11	0.66	0.71	0.60	0.68	0.65	0.54	0.64	0.76	0.68	0.62	0.69	0.70	0.60	0.79	0.75	0.76	0.69	0.68
12	0.67	0.72	0.56	0.68	0.54	0.52	0.60	0.75	0.67	0.67	0.70	0.71	0.61	0.69	0.69	0.64	0.64	0.65

Items:
1 to find out where to get professional help when you are ill?
2 to understand information about what to do in a medical emergency?
3 to judge the advantages and disadvantages of different treatment options?
4 to act on advice from your doctor or pharmacist?
5 to find information on how to handle mental health problems?
6 to understand information about recommended health screenings or examinations?
7 to judge if information on unhealthy habits, such as smoking, low physical activity or drinking too much alcohol, are reliable?
8 to decide how you can protect yourself from illness using information from the mass media?
9 to find information on healthy life styles such as physical exercise, healthy food or nutrition?
10 to understand advice concerning your health from family or friends?
11 to judge how your housing conditions may affect your health and well-being?
12 to make decisions to improve your health and well-being?
