# Supplementary material for: Measuring Comprehensive, General Health Literacy in the General Adult Population: The Development and Validation of the HLS19-Q12 Instrument in Seventeen Countries
Source: Int J Environ Res Public Health. 2022 Oct 29;19(21):14129. doi: 10.3390/ijerph192114129 (PMC9659295; doi:10.3390/ijerph192114129)
Supplement: Supplementary file 1 [file ijerph-19-14129-s001.zip › Supplementary_TableS3.pdf]

Table S3. Socio-demographic variables included in the regression models.

	AT	BE	BG	CH	CZ	DE	DK	FR	HU	IE	IL	IT	NO	PT	RU	SI	SK
**Gender**																	
Male	1449	496	411	1230	780	1052	1708	973	559	2189	631	1682	1448	604	2565	1675	1028
Female	1518	504	454	1269	819	1085	1894	1030	636	2294	684	1818	1407	643	3095	1685	1117
Missing	0	0	0	3	0	6	0	0	0	4	0	0	0	0	0	0	0
**Age Groups**																	
18-25	354	90	99	274	150	216	366	260	131	517	178	329	360	165	553	317	276
26-35	498	207	115	425	320	324	507	353	228	741	265	453	496	181	1200	505	376
36-45	474	142	153	423	263	310	505	379	195	938	266	559	473	255	1046	606	377
46-55	584	221	134	464	248	406	655	391	223	804	196	674	491	232	900	598	341
56-65	448	177	170	382	274	361	611	365	184	651	197	570	411	220	1029	571	328
66-75	333	135	142	288	264	256	563	256	154	623	180	460	327	127	567	423	276
$\geqslant 76$	271	28	45	243	81	251	394	0	82	213	34	455	213	48	365	340	163
Missing	5	0	7	4	0	19	0	0	0	0	0	0	84	19	0	0	8
**Highest level of education**																	
Lower secondary or below	557	30	80	334	711	235	353	72	608	1224	75	1510	263	505	366	1139	394
Higher secondary	1438	117	193	1175	500	1012	315	282	307	959	364	1118	811	380	630	1260	800
Above secondary	972	841	586	985	387	853	2930	1649	280	2287	876	872	1759	362	4643	961	947
Missing	0	12	6	8	1	42	5	0	0	17	0	0	21	0	21	0	4
**Financial Deprivation**																	
None	2130	510	471	1317	857	1352	2872	1263	490	2669	569	1505	2476	637	2081	1415	880
Some	376	124	172	404	299	228	364	344	241	750	250	525	199	202	963	534	283
Considerable	223	116	79	321	231	140	208	193	150	524	220	720	67	79	849	469	403
Severe	115	250	113	414	191	218	131	203	242	498	155	551	42	250	1427	835	374
Missing	122	0	30	47	20	204	28	0	72	47	122	199	72	79	340	108	205
**Self-perceived level in society**																	
1 lowest	5	3	18	31	26	10	46	10	18	68	15	54	15	6	73	45	13
2	18	17	15	37	31	33	54	44	42	59	24	66	41	16	106	89	29
3	73	34	42	94	72	112	161	128	97	142	33	168	73	76	374	275	113
4	138	52	60	176	130	245	195	225	169	276	97	310	124	210	718	387	168
5	811	117	181	558	528	417	652	508	416	1283	228	675	579	358	1411	1006	531
6	504	206	146	455	307	502	687	487	246	912	247	1050	527	325	991	662	441
7	687	322	160	412	304	442	926	391	128	920	316	801	787	178	1006	551	336
8	422	203	90	234	141	271	619	167	43	479	185	246	451	71	518	227	231
9	71	41	39	48	19	45	162	30	10	82	47	39	102	5	197	29	61
10 highest	58	5	16	29	31	9	89	12	12	173	54	29	79	2	131	23	45
Missing	181	0	99	429	11	57	11	0	15	94	69	63	77	0	134	66	177
**Total N**	2967	1000	865	2502	1599	2143	3602	2003	1195	4487	1315	3500	2855	1247	5660	3360	2145
